# Heritable *GATA2* mutations associated with familial AML-MDS: a case report and review of literature

**DOI:** 10.1186/1756-8722-7-36

**Published:** 2014-04-22

**Authors:** Juehua Gao, Ryan D Gentzler, Andrew E Timms, Marshall S Horwitz, Olga Frankfurt, Jessica K Altman, LoAnn C Peterson

**Affiliations:** 1 Department of Pathology, Northwestern University Feinberg School of Medicine, 251 E. Huron Street, Chicago, IL 60611, USA; 2 Department of Internal Medicine, Division of Hematology and Oncology, Northwestern University Feinberg School of Medicine, 251 E. Huron Street, Chicago, IL 60611, USA; 3 Department of Pathology, University of Washington, Seattle, WA 98195, USA; 4 Present affiliation: Seattle Children’s Research Institute, 1900 9th Ave, Seattle, WA 98101, USA

**Keywords:** GATA2, Familial acute myeloid leukemia-myelodysplastic syndrome

## Abstract

A 50-year-old woman was diagnosed with acute myeloid leukemia (AML). She has history of thrombocytopenia for 25 years and a significant family history of thrombocytopenia, affecting her mother, siblings and their children, as well as her own children. Both her mother and maternal aunt died from myelodysplastic syndrome (MDS). Additional genetic analysis was performed and identified two heterozygous missence mutations in the second zinc finger domain of GATA2 gene (p.Thr358Lys, and p.Leu359Val), occurring in cis on the same allele. Given the patient’s family history and clinical manifestation, this was interpreted as an acute myeloid leukemia with heritable GATA2 mutations associated with familial AML-MDS. Germline GATA2 mutations are involved in a group of complex syndromes with overlapping clinical features of immune deficiency, lymphedema and propensity to acute myeloid leukemia or myelodysplastic syndrome (AML-MDS). Here we reported a case of familial AML-MDS with two novel GATA2 mutations. This case illustrates the importance of recognizing the clinical features for this rare category of AML-MDS and performing the appropriate molecular testing. The diagnosis of heritable gene mutations associated familial AML-MDS has significant clinical implication for the patients and affected families. Clinical trials are available to further investigate the role of allogeneic hematopoietic stem cell transplant in managing these patients.

## Background

MDS and AML are mostly sporadic hematopoetic malignancies typically affecting older patients. Familial occurrence of MDS or AML is rare, and most of these cases arise in the setting of genetic syndromes associated with increased risks of developing AML or MDS, including several inherited bone marrow failure syndrome, such as Diamond-Blackfan anemia, severe congenital neutropenia, Shwachman-Diamond syndrome, and dyskeratosis congenital. Rare familial cases of MDS and AML have been reported in families without congenital syndromes who carry germ line predisposing mutations. Examination of families with MDS and AML has led to the detection of several inherited mutations in RUNX1 or CEBPA, and more recently GATA2. Here we reported a case of familial MDS-AML with two novel GATA2 mutations.

## Case report

A 50-year-old Caucasian woman with a 25 year history of thrombocytopenia presented to the emergency department with worsening cough and high fever and was diagnosed with bilateral multilobar Legionella pneumonia. Her clinical condition deteriorated rapidly into multiorgan failure, requiring pressors, hemodialysis, and artificial ventilation. She was noted to have circulating blasts in the peripheral blood.

She had a significant family history of thrombocytopenia, affecting her mother, siblings and their children, as well as her own children. Both her mother and maternal aunt died from MDS.

**Figure 1:**
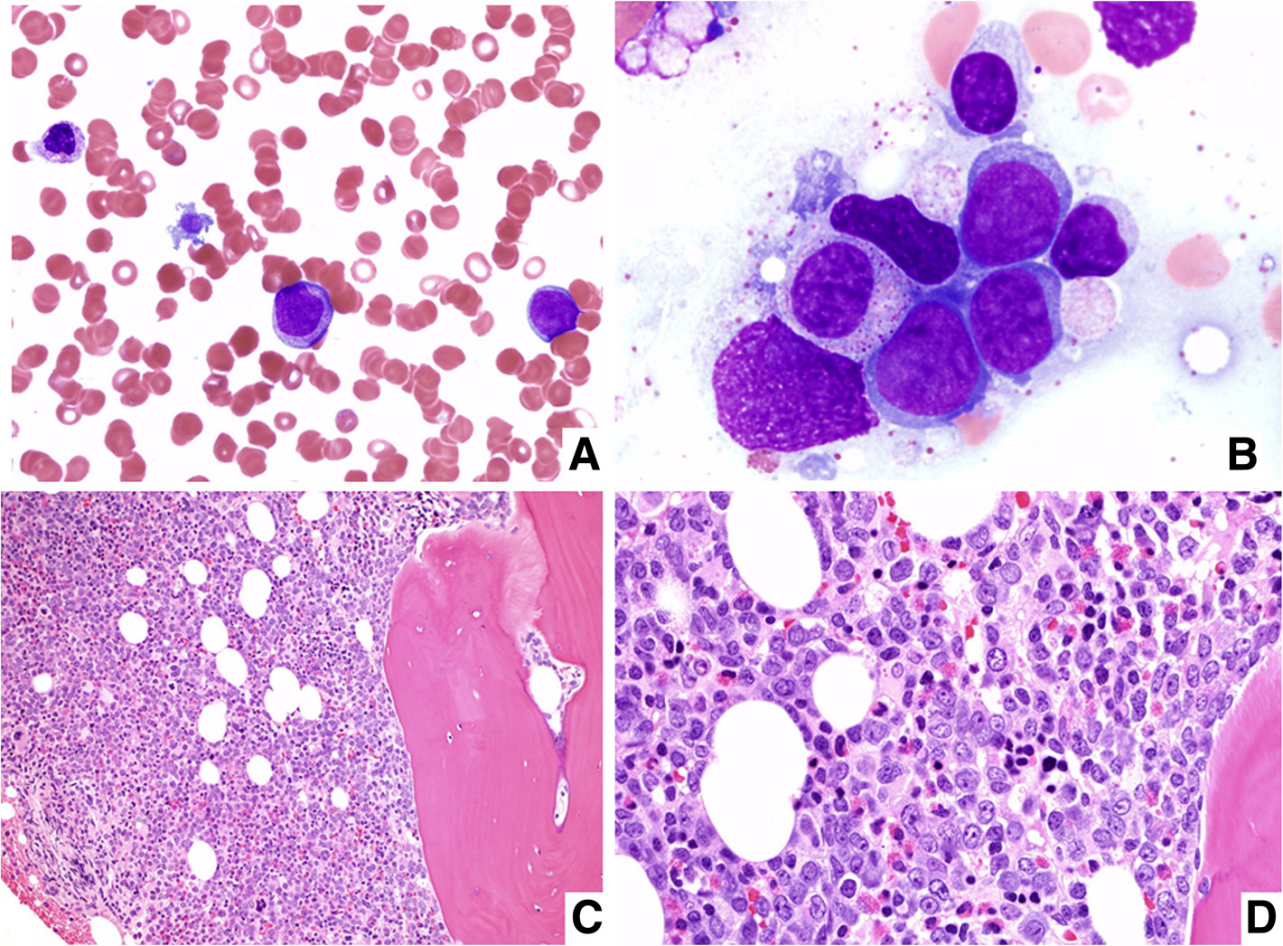
**Morphological findings of familial AML-MDS with inherited GATA2 mutations. (A)** Peripheral blood smear revealed increased number of blasts, occasional dysplastic neutrophils with hyposegmented nuclei and platelets with abnormal morphology (Wright-Giemsa, ×400). **(B)** Bone marrow aspirate smears contain occasional blasts with fine chromatin, round nuclei and scant cytoplasm (Wright-Giemsa, ×1000). **(C)** and **(D)** The bone marrow core biopsy sections showed a markedly hypercelluar bone marrow with significantly increased numbers of blasts **(C**: Hematoxylin and eosin, ×200, **D**: Hematoxylin and eosin, ×600).

**Figure 2:**
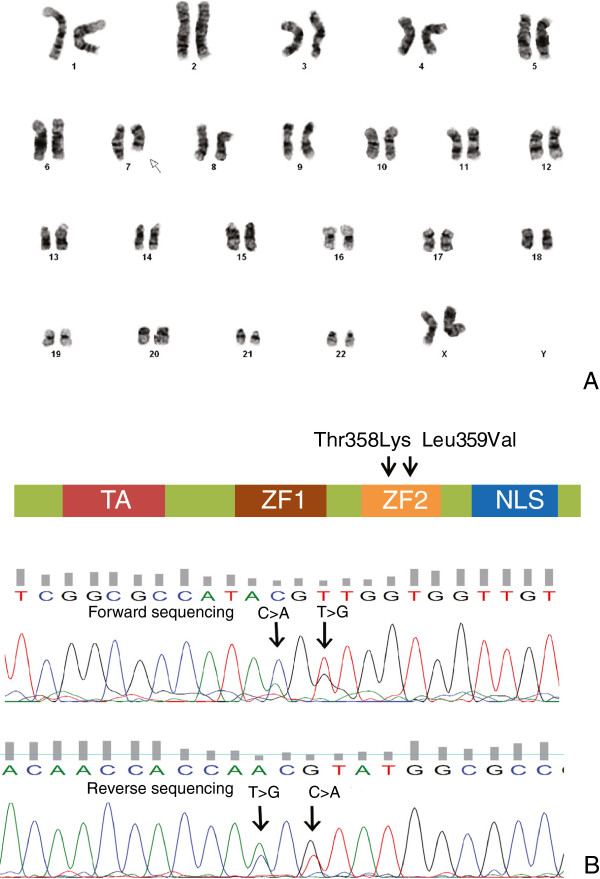
**Cytogenetic and molecular findings of familial AML-MDS with inherited GATA2 mutations. (A)** Cytogenetic analysis performed on fresh bone marrow aspirate revealed all 20 cells with a deletion of long arm of chromosome 7, i.e. 46,XX,del(7)(q22q36)[20]. **(B)** To screen GATA2 for known mutations, PCR products were amplified by either Qiagen Taq or by Roche GC rich PCR kit using primers listed in table below. PCR products were Sanger sequencing using the same primers with Big Dye chemistry (ABI) on a 3730xl DNA Analyzer (ABI). Bidirectional Sanger sequencing revealed 2 heterozygous mutations in 2^nd^ zinc finger domain of *GATA2* gene, p. Thr358Lys (c.1074 C > A) and p.Leu359Val (c.1076 T > G). To differentiate whether the mutations were cis or trans, PCR products were cloned into a pCI vector (Promega) and 8 clones were sequenced. Of the 8 clones, 3 failed to produce sequence information, 2 contained both wild type alleles and 3 contained both mutant alleles.

Her peripheral blood revealed normochromic normocytic anemia with hemoglobin of 9.5 g/dL, thrombocytopenia (55,000/uL), white blood cells of 8700/μL, including 25% of blasts. Morphologic review of the peripheral blood smear revealed scattered blasts with high nuclear to cytoplasmic ratio, some with cytoplasmic granules. Occasional dysplastic neutrophils with hyposegmented nuclei were also noted. Some of the platelets were large and irregular (Figure 1A). Bone marrow aspirate smears were diluted by peripheral blood, but there were blasts with fine chromatin, round nuclei and scant cytoplasm (Figure 1B). The particle clot and core biopsy sections showed markedly hypercelluar marrow with increased blasts (Figure 1C, D). Flow cytometric immunophenotyping performed on the bone marrow aspirate revealed a myeloid blast population that was partial CD34+, CD117+, with dim expression of myeloperoxidase, CD13 and CD33, and aberrant expression of CD7. The flow cytometric results support the diagnosis of acute myeloid leukemia. Cytogenetic analysis performed on fresh bone marrow aspirate revealed 46,XX,del(7)(q22q36)[20] (Figure 2A). Based on the morphologic and cytogenetic findings, the patient was diagnosed with acute myeloid leukemia with myelodysplasia related changes. Molecular tests for *FLT3 ITD*, *FLT3 D835* and *NPM1* were performed on DNA extracted from fresh bone marrow aspirates using an automated nucleic extraction instrument QIAsymphony followed by previously described methodology [1,2]. *CEBPA* and *KIT* mutational analyses were sent to Blood Center of Wisconsin and Mayo Medical Laboratory respectively. These tests did not identify mutations involving *FLT3 ITD, FLT3 D835*, *NPM1*, *CEBPA*, and *KIT* genes. Given her significant family history of thrombocytopenia and MDS, there was a concern for a heritable mutation as a predisposition gene for familial MDS-AML. Additional genetic analysis of *RUNX1* as previously described did not reveal any mutations [3]. Sanger sequencing was performed to screen the known mutation of *GATA2* and identified two heterozygous missence mutations in the second zinc finger domain of *GATA2* gene (p.Thr358Lys, and p.Leu359Val) (Figure 2B, Table 1). The two mutations occur in cis on the same allele, as determined by subcloning and sequencing individual clones. Given the patient’s family history and clinical manifestation, this was interpreted as an acute myeloid leukemia with heritable *GATA2* mutations associated with familial AML-MDS. Patient required multiple cycles of chemotherapy to achieve a remission and eventually underwent a double umbilical cord hematopoietic stem cell transplant (HSCT) with reduced intensity conditioning. Unfortunately, she died 6 months later due the transplant-related complications while in complete morphologic, cytogenetic, and molecular remission, demonstrating 100% single cord donor chimerism. Although it was recommended to confirm the germ line nature of the *GATA2* mutation by submitting additional material such as a skin biopsy or a buccal swab for germline *GATA2* testing, it was not performed due to the patient’s poor condition from persistent chronic infection and respiratory failure. Other family members declined testing for *GATA2* mutations.

**Table 1:** Primers used in GATA2 sequencing

**Exon**	**Forward primer**	**Reverse primer**	**PCR size**
e1	tcctttcgttttgagccttg	ttttcagcagctcgattcct	552
e2	ctggttctgggagtcgtgat	catcttcatgctctccgtca	542
“”	ggaggcccactctctgtgta	cctctcccaagtcacagctc	548
e3	agaccctctcgtccctcttc	agacgaccccaactgacatc	464
e4	cttgcaatcccgttgattct	agccaagctggatattgtgg	466
e5	ctatgaaggtcgggcacaat	gtcctcgacgtccatctgtt	457

## Discussion

*GATA2* is a transcription factor crucial for hematopoietic differentiation and lymphatic formation. The biologic function of *GATA2* in hematopoiesis is diverse through interactions with various transcriptional factors and cofactors. *GATA2* plays an essential role in maintaining the proliferation and survival of early hematopoietic cells, as well as preferential differentiation to erythroid or megakaryocytic lineages [4,5]. Expression of *GATA2* is significantly higher in AML compared to normal bone marrow, and is an adverse indicator of prognosis [6,7]. Mutations involving *GATA2* coding sequence are not common in sporadic AML cases, and are frequently associated with a more specific subgroup of AML with normal cytogenetics and biallelic *CEBPA* mutations. In these cases, somatic mutation of *GATA2* is likely a secondary event in the leukemogenesis [6,8-10]. However, germline *GATA2* mutations have a very different oncogenic role. Germline *GATA2* mutations are involved in a group of complex syndromes with overlapping clinical features, including a rare genetic disorder called MonoMAC, Emberger syndrome and familial AML following MDS. These diverse syndromes may reflect different clinical manifestations of the common underlying defect of *GATA2* deficiency. MonoMac is a complex congenital immunodeficiency characterized by persistent and profound peripheral monocytopenia, B- and NK-cell lymphocytopenia, near absence of dendritic cells and increased susceptibility to mycobacterium or papilloma virus infections [11-13]. Emberger syndrome is characterized by primary lymphedema inherited in an autosomal dominant pattern [14,15]. Both syndromes are associated with a predisposition to acute myeloid leukemia and myelodysplastic syndrome. *GATA2* has recently been recognized as a MDS-AML predisposition gene, in addition to the previously reported *RUNX1* and *CEBPA*. Since the first report of 4 families of heritable *GATA2* mutations associated with familial AML-MDS by Hahn *et al.* in 2011, [10] there have been more than a dozen pedigrees reported in the literature (Table 2). Studies of these families provide significant insights on the genetic and clinical features of this rare form of AML-MDS.

**Table 2:** **Reported pedigrees of germline *GATA2 *** **deficiency associated with familial AML-MDS**

**Authors**	**Pedigrees**	**Mutation**	**Clinical features**
Hahn et al. [10]	4 pedigrees	p.Thr354Met, p.Thr355del	Familial AML-MDS
Bodor et al. [16]	1 pedigree	p.Thr354Met	Familial AML-MDS
Holm et al. [17]	4 pedigrees	p.Leu105ProfsX15, p.Pro41Ala, p.Arg396Glu, p.Thr354Met	Familial AML-MDS, lymphedema, skin cancer
Pasquet et al. [18]	7 pedigrees	p.Arg396Gln, p.Arg204X, p.Glu224X, p.Arg330X, p.Ala372Thr, p.Met388Val, and a 61 kb deletion of the *GATA2* locus	Chronic neutropenia and evolution to AML-MDS
Kazenwadel et al. [19]	2 pedigrees	p.Thr354Met, p.Met1del290	Familial MDS, MonoMac

Patients with familial AML-MDS are younger at presentation than individuals with sporadic disease. Most of the families manifest an unusual family history of more than one first-degree relative with AML-MDS, consistent with a pattern of autosomal dominant inheritance. But there is clearly heterogeneity in the clinical features from the cases reported [10,17]. The patients may or may not have precedent hematologic abnormalities, and the onset age of AML-MDS in affected family members are variable. Cases described also demonstrate a spectrum with different morphologic subtypes and variable cytogenetic abnormalities, including most frequently monosomy 7, but also trisomy 8, and trisomy 21 [10].

The mutations associated with *GATA2* also demonstrate marked genetic heterogeneity [17]. The GATA2 protein contains a transactivation domain in the N-terminus and two highly conserved zinc finger (ZF) domains. Mutations previously described are highly heterogeneous ranging from single base substitutions, insertions and deletions, and are present throughout the gene. There are two major classes of mutations involving *GATA2* reported [20]. Multiple studies described N-terminal frameshift mutations cause premature terminations and result in a nonfunctional protein lacking most of the C-terminal [21]. The mutations in C-terminal zinc finger domains are predicted to cause significant structural alterations critical for interaction with DNA, other transcription factors and cofactors, causing more variable phenotypic consequences [17]. The mutations reported from affected families are variable and present throughout the *GATA2* gene (Table 2). The majority of these mutations are missence mutation, with the p.Thr354Met mutation being the most frequently mutated in the reported families [10,16,17,19].

The p.Thr358Lys and p.Leu359Val reported in this case have not been seen as a germline event in familial AML/MDS in the literature. The p.Thr358Lys mutation is listed on the NHLBI Exome Variant Server (http://evs.gs.washington.edu/EVS/) at a frequency of 1/4000 individuals without available history, but may include some with the potential to develop MDS. It is not a known genetic variant in the single nucleotide polymorphism site in the Single Nucleotide Polymorphism Database (dbSNP). The p.Leu359Val has been exclusively identified in myeloid transformation of chronic myeloid leukemia [22]. Zhang *et al.* reported a p.Leu359Val in 8 of 85 cases of CML in blast crisis and associated with myelomonoblastic features [22]. Further studies demonstrated p.Leu359Val has a gain of function effect with increased transactivation activity of *GATA2* but also enhanced its inhibitory effects on the activity of PU.1, a major transcription factor for myeloid cell differentiation, via aberrant protein–protein interaction [22]. Although neither mutation has not been reported in familial MDS-AML in the literature, nearby residues in the second zinc finger have been found to be frequently mutated, so they could very well be novel germline mutations. These two mutations come from the same parent as both occur on the same allele, but it is unclear whether one of them is sufficient to cause the disease phenotype.

*GATA2* is probably an important predisposing mutation but secondary genetic events are required for the development of overt malignant disease. Acquiring other genetic abnormalities may also affect the phenotype in addition to the *GATA2* mutation status. The secondary genetic events in patients with heritable *GATA2* mutations associated with familial AML-MDS are still mostly unknown, but several cases have been reported in which acquired *ASXL1* mutations are common in patients with inherited *GATA2* mutations. West *et al.* reported somatic, heterozygous *ASXL1* mutations were identified in 14 of 48 (29%) patients with inherited *GATA2* deficiency and that correlated with myeloid transformation [23]. Partial or complete loss of chromosome 7 has been reported as a common cytogenetic finding in multiple pedigrees with familial AML-MDS [24,25]. Chromosome 7 abnormalities are not inherited but likely represent a recurring secondary event in leukemogenesis and are frequently linked to the development of AML-MDS.

Heritable *GATA2* mutations associated with familial myelodysplastic syndrome and acute myeloid leukemia have only been described recently. There is no clear correlation between the genotype and clinical outcome. Based on limited cases reported in the literature, the affected individuals usually have a poor outcome unless successfully transplanted [10,16]. A recent study reported six patients who underwent allogeneic stem cell transplant for *GATA2* deficiency had excellent outcomes except one who died from infection [26]. Other anecdotal reports implied a less ideal outcome [16]. It is likely other secondary genetic event such as *ASXL* mutation and loss of chromosome 7 confer a poorer prognosis. Due to the rarity of affected pedigrees, current clinical management and guidelines for risk stratification and treatment are mostly based on expert opinion. However, there are clinical trials available to elucidate the risk stratification and determine those who might benefit from early intervention such as allogeneic bone marrow transplantation (ClinicalTrials.gov: NCT01861106). Another important feature often complicating the clinical course of patients with AML is infection, particularly intracellular organisms such as atypical mycobacteria and viruses, as a result of underlying immunodeficiency due to cytopenias from AML-MDS and/or chemotherapy. Serious infections are often the causes of death, as seen in our patient.

It is import to identify familial cases of AML-MDS and test the heritable mutations. A complete clinical and family history is a clue to recognizing patients with an inherited predisposition to myeloid neoplasm. Germline testing should be considered in a family with more than one close relatives affected by AML-MDS or in patients with early onset disease. A recent study based on large population based registry data did not show that relatives of patients with AML-MDS are at increased risk of hematologic tumor, but there is a significant increased risk of AML-MDS and other myeloid malignancies among first degree relatives of patients diagnosed at younger than age 21 years [27]. This suggests that young age at the onset of disease is probably the most useful indicator to look for inherited factors in developing AML-MDS. Germline testing should be performed in specimens containing only the nonleukemic cells such as skin fibroblasts. Buccal swab or saliva samples are acceptable; though these may contain lymphocytes derived from hematopoietic stem cells. Given the genetic heterogeneity in cases of germline *GATA2* mutation, sequencing of the entire coding region of *GATA2* is needed. With the anticipated rapid incorporation of next-generation sequencing into clinical practice, testing genes involved in inherited predisposition syndromes is becoming increasingly available.

The detection of an underlying germ line mutation has significant implications for clinical practice. Due to the poor outcomes in the reported AML cases with *GATA2* mutations, aggressive and early intervention such as allogeneic stem cell transplant should be considered. Family members with identified germline mutations should be avoided as stem cell donors. Although there is no direct data, family members under consideration for being HSCT donors should be tested to exclude mutations in the same predisposition gene, due to the theoretical risk of developing AML in the future from the graft with the same mutation. Genetic counselling should be offered to the family members, as germ line testing for these types of mutations has ethical and psychological implications for those being tested and their families. We would recommend mutation carriers in the family to have CBC and a baseline bone marrow biopsy to evaluate for occult myeloid neoplasm. Close clinical follow up for affected family members is particularly important. For affected mutation carriers without morphologic evidence of myeloid neoplasm , whether and when to consider the HSCT is not known. Identification of prognostic markers which may help select patients at high risk of developing AML-MDS will be particularly helpful.

## Conclusion

In addition to *RUNX1* and *CEBPA*, *GATA2* gene mutations have only been recently reported involved in familial AML-MDS. Here we reported two novel *GATA2* mutations occurring in cis on the same allele associated with a case of familial AML-MDS. Heritable gene mutations as a predisposition gene to AML-MDS are likely under recognized, but have significant implication in managing the patients and the affected families. It is important to recognize this rare entity, be familiar with the clinical features, and seek appropriate laboratory testing when there is a clinical concern. With the rapid availability of genetic testing, particularly in the era of next generation sequencing, accumulating cases will provide insights to the molecular events in leukemogenesis, and clinical trials aimed at identifying the appropriate treatment for specific molecular subsets of AML will enhance our understanding of these heterogeneous diseases and may benefit patients and their families.

## Consent

Written informed consent was obtained from the patient’s next kin for publication of this case report and any accompanying images. A copy of the written consent is available for review by the Editor-in-Chief of this journal.

## Competing interests

The authors declare that they have no competing interests.

## Authors’ contributions

JG, reviewed the literature and wrote the paper. RDG, OF and JKA: treated the patient and collected the data. AET, MSH, performed the molecular analysis. LCP, carried out critical interpretations. All authors read and approved the final manuscript.
