# Erratum: Heritable GATA2 mutations associated with familial AML-MDS: a case report and review of literature

**DOI:** 10.1186/s13045-015-0228-z

**Published:** 2015-12-29

**Authors:** Juehua Gao, Ryan D. Gentzler, Andrew E. Timms, Marshall S. Horwitz, Olga Frankfurt, Jessica K. Altman, LoAnn C. Peterson

**Affiliations:** Department of Pathology, Northwestern University Feinberg School of Medicine, Feinberg 7-209A, 251 E. Huron Street, Chicago, IL 60611 USA; Division of Hematology and Oncology, Department of Internal Medicine, Northwestern University Feinberg School of Medicine, 251 E. Huron Street, Chicago, IL 60611 USA; Department of Pathology, University of Washington, Seattle, WA 98195 USA; Present address: Seattle Children’s Research Institute, Seattle, WA 98101 USA

After the publication of this work [1], it was brought to our attention that the correct annotation at the protein level for one of the two reported GATA2 mutations is T358N (p.Thr358Asn), but not T358K (p. Thr358Lys), as the forward and reverse sequencing in Fig. 2b were swapped leading to the previous incorrect annotation.
